# Inequalities in the practice of physical activity in the city of São Paulo between 2003 and 2015: Evidence from a population-based study among older adults

**DOI:** 10.1186/s13690-025-01630-3

**Published:** 2025-08-11

**Authors:** Bruno Holanda Ferreira, Camila Nascimento Monteiro, Tatiane Kosimenko Ferrari Figueiredo, Margareth Guimarães Lima, Chester Luiz Galvão Cesar, Moisés Goldbaum, Olinda do Carmo Luiz

**Affiliations:** 1 Departamento de Medicina Preventiva, Faculdade de Medicina da Universidade de São Paulo, Av. Dr. Arnaldo, 455 - sala 2172, São Paulo, SP 01246-903 Brazil; 2 Hospital Sírio-Libanês, São Paulo, SP Brazil; 3 Departamento de Epidemiologia, Faculdade de Saúde Pública, Universidade de São Paulo, São Paulo, SP Brazil; 4 Departamento de Saúde Coletiva, Universidade Estadual de Campinas, São Paulo, Brazil

**Keywords:** Active aging, Public health, Exercise, Health risk behaviors, Health surveys, Envelhecimento ativo, Saúde pública, Exercício físico, Comportamentos de risco à saúde, Inquéritos de saúde

## Abstract

**Background:**

Regular physical activity is essential for the health and quality of life of older adults, helping to prevent non-communicable diseases and maintain autonomy. However, sociodemographic factors influence physical activity levels, leading to disparities in access and adherence. This study aimed to analyze changes in the frequency of physical activity and sociodemographic inequalities among older adults living in Brazil, using data from two health surveys.

**Methods:**

The information on older adults (60 years or older) was obtained from two Health Surveys (ISA-Capital) conducted in the city of São Paulo, Brazil, in 2003 and 2015. The variables analyzed included sociodemographic characteristics and physical activity levels, considering the combined domains of leisure-time and transportation. Prevalence and 95% Confidence Intervals (95% CI) were calculated, and comparisons were made via crude and adjusted Prevalence Ratios (PR) by Poisson regression.

**Results:**

Between 2003 and 2015, the prevalence of sufficient physical activity increased from 20.5% to 30.1%, representing a percentage change of 46.8%. When stratified by sociodemographic characteristics, the 2015 survey showed a significant increase in older adults with a sufficient level of physical activity concentrated among individuals aged 60–69 years (PR = 1.43; 95%CI 1.10–1.86), male (PR = 1.45; 95%CI 1.10–1.91), with partner (PR = 1.42; 95%CI 1.08–1.88), with up to 3 years of education (PR = 1.53; 95%CI 1.11–2.09), and who self-identified as race/skin color black or brown (PR = 1.70; 95%CI 1.12–2.59), when compared to their respective counterparts in the 2003 survey.

**Conclusions:**

Overall, over 12 years, the prevalence of older adults achieving sufficient physical activity levels increased, although unevenly, highlighting the need for policies and interventions to promote more significant equity in access to physical activity opportunities.

**Significance/Implications:**

These findings highlight the persistent inequalities in physical activity, emphasizing the need for targeted policies and interventions to ensure equitable access to physical activity opportunities among older adults.

## Resumo

**Introdução:**

A prática regular de atividade física é essencial para a saúde e a qualidade de vida de pessoas idosas, contribuindo para a prevenção de doenças crônicas não transmissíveis e manutenção da autonomia. No entanto, fatores sociodemográficos influenciam os níveis de atividade física, gerando desigualdades no acesso e na adesão. Este estudo teve como objetivo analisar as mudanças na frequência da prática de atividade física e as desigualdades sociodemográficas entre idosos residentes no Brasil, utilizando dados de dois inquéritos de saúde.

**Métodos:**

As informações de idosos (60 anos ou mais) foram obtidas a partir de dois Inquéritos de Saúde (ISA-Capital), realizados na cidade de São Paulo, Brasil, em 2003 e 2015. As variáveis analisadas incluíram características sociodemográficas e níveis de atividade física, considerando os domínios combinados de lazer e deslocamento. Foram calculados as prevalências e os Intervalos de Confiança de 95% (IC95%), com comparações por meio de Razões de Prevalência (RP) brutas e ajustadas, obtidas pela análise de regressão de Poisson.

**Resultados:**

Entre 2003 e 2015, a prevalência de atividade física em nível suficiente aumentou de 20,5% para 30,1%, o que corresponde a um aumento percentual de 46,8%. Quando estratificado por características sociodemográficas, o inquérito de 2015 identificou aumento significativo de idosos com nível de atividade física suficiente entre aqueles na faixa etária de 60 a 69 anos (RP = 1,43; IC95% 1,10–1,86), sexo masculino (RP = 1,45; IC95% 1,10–1,91), com companheiro(a) (RP = 1,42; IC95% 1,08–1,88), com até 3 anos de escolaridade (RP = 1,53; IC95% 1,11–2,09) e que se autodeclararam pretos ou pardos quanto à raça/cor da pele (RP = 1,70; IC95% 1,12–2,59), quando comparados aos seus respectivos pares do inquérito de 2003.

**Conclusões:**

De forma geral, ao longo de 12 anos, a prevalência de idosos que atingiram níveis suficientes de atividade física aumentou, embora de maneira desigual, o que ressalta a necessidade de políticas e intervenções que promovam maior equidade no acesso às oportunidades de prática de atividade física.

**Significado/Implicações:**

Os achados ressaltam as persistentes desigualdades na prática de atividade física, enfatizando a importância de políticas e intervenções direcionadas para garantir o acesso equitativo entre a população idosa.

**Table:** 

Text box 1. Contributions to the literature	
• This study explores how sociodemographic disparities influence physical activity trends among older adults, highlighting persistent inequalities. • By analyzing two waves of a population-based health survey, this research provides long-term evidence on the evolution of physical activity patterns in a rapidly aging urban population. • The findings highlight the need for targeted public health interventions to reduce disparities in physical activity levels, particularly among older adults over 70 years old, without a partner, females, different educational levels, and those from racially diverse backgrounds. • This study underscores the importance of considering social determinants of health when designing policies to promote active aging and prevent non-communicable diseases.	

## Introduction

The aging of the global population due to advancements in the treatment and management of various diseases poses a growing challenge to public health and healthcare systems. It is estimated that by 2050, approximately 22% of the world’s population will be 60 years or older [1], whereas in Brazil, this proportion is expected to reach 13,6% by 2030 [2]. These demographic changes have sparked increasing scientific interest in investigating factors associated with health behaviors among older adults, given that lifestyle is one of the primary determinants of healthy aging [3].

Health surveys play a crucial role in monitoring health behaviors among older adults. They have been instrumental in tracking trends in risk behaviors such as smoking, alcohol consumption, unhealthy eating, and physical inactivity [4]. Global evidence indicates a reduction in some of these unhealthy behaviors [5–8]. However, inequalities and barriers faced by different sociodemographic groups in the prevalence of these behaviors have intensified in several countries [9–11], underscoring the importance of monitoring and analyzing them at local levels.

The World Health Organization (WHO) recommends that all countries conduct surveillance of risk factors for noncommunicable chronic diseases, including physical activity, across all age groups. The WHO also highlights the importance of publishing disaggregated data reports to track trends among subpopulations—such as by gender, age, ethnicity, and socioeconomic status—to identify and monitor efforts to reduce inequalities [9].

In Brazil, monitoring behavioral risk factors has proven essential. In recent years, Brazilian studies have reported a stable prevalence of individuals reporting healthy eating [12], smoking habits [13], alcohol consumption [13], and physical activity in the transportation domain [14]. Conversely, an increase in the prevalence of physical activity in the leisure-time domain has been reported [15]. Although national and local surveys indicate an increase in leisure-time physical activity in Brazil in recent decades, this overall progress may obscure persistent or even widening inequalities between different social groups. Among older adults—a population with specific health vulnerabilities and barriers to physical activity—understanding trends in physical activity across sociodemographic strata is crucial to identify whether public health strategies have reached all groups equitably.

Given the importance of engaging in 150 min or more of physical activity per week to maintain functional capacity, physical fitness, and manage chronic diseases [9], public health officials must prioritize policies that encourage physical activity as part of their strategies. Such efforts aim not only to improve population health but also to reduce healthcare-related costs. This study aims to analyze trends in physical activity among older adults residing in the city of São Paulo, Brazil, and to examine whether sociodemographic inequalities in physical activity have changed between 2003 and 2015.

## Methods

### Sampling methods and data sources

This serial time-series population-based study utilizes primary source data from individuals aged 60 years or older, collected from the São Paulo Health Surveys (ISA-Capital) conducted in 2003 and 2015. The study sample represents the urban population of city of São Paulo, a city located in southeastern Brazil. São Paulo has over 12 million residents, approximately 11.6% of whom are aged 60 and above. It is the most populous and economically significant city in Brazil, and the fourth-largest metropolis in the world in terms of population.

### ISA-Capital 2003

The 2003 edition of ISA-Capital 2003 sampling plan used a cluster sampling approach, selecting 60 of the 264 census tracts in the city of São Paulo. The tracts were weighted to compensate for different probabilities of selection, considering the proportion of household heads with varying levels of education. One-third of the sample was drawn from each stratum, resulting in 420 planned interviews per domain. This allowed for estimating a prevalence of 0.5 with a sampling error of 0.06, a 5% significance level, and a design effect of 1.5, accounting for potential losses. 872 individuals aged 60 years or older of both sexes were interviewed, representing approximately 982,511 residents living in private, permanent homes in the city of São Paulo in 2003. A detailed description of the ISA-Capital 2003 sampling plan is available in another publication [16].

### ISA-Capital 2015

The sampling was conducted using a complex and probabilistic methodology involving a random selection of census tracts and households. Two domains were considered: geographic and demographic. These included regional health districts (Central-West, East, North, Southeast, and South) and demographic districts (male and female older adults). The sample consisted of 980 individuals, with 162 to 234 planned interviews per district. This allowed for the estimation of proportions of 0.5 with a sampling error of 0.10, a 95% confidence level, and a design effect of 1.5. All the selected households were visited at least three times, resulting in a household response rate of 76% and an individual response rate of 74%. Post-stratification weights were used to adjust the sample distribution to the population estimates by sex and age group, reducing potential response bias. 1019 individuals aged 60 years or older of both sexes were interviewed, representing approximately 1,604,869 residents living in private, permanent homes in the city of São Paulo in 2015. A detailed description of the ISA-Capital 2015 sampling plan is available in another publication [17].

In both surveys, homeless individuals and institutionalized residents were excluded. Data were collected via questionnaires administered by trained interviewers and completed directly by male and female residents aged 60 years or older. The questionnaires were organized into thematic blocks, with most questions being closed-ended with predefined options. Informed consent was obtained from all participants after a complete explanation of the study’s purpose and procedures was provided, and all participants signed a free and informed consent form. The study complies with Resolution No. 466/12 of the Brazilian National Health Council (CNS) and was approved by the Ethics Committee of the Faculdade de Saúde Pública da Universidade de São Paulo, São Paulo, Brazil (Protocol: 719.661/2014).

### Principal variables

Physical activity was the dependent variable based on the sum of physical activities performed in the leisure-time and transportation domains [18]. Information was collected via the long version of the International Physical Activity Questionnaire (IPAQ), a validated tool widely used in other studies involving ISA-Capital data [18, 19]. The questions addressed weekly frequency and total daily time of physical activity, including inquiries such as:"During a typical week, on how many days did you engage in vigorous physical activity for at least 10 min?"The number of responses ranged from 1 to 7 days. Another question asked:"How much time do you usually spend doing vigorous physical activity on one of these days?".

The scores were converted into minutes per week according to international IPAQ guidelines (www.ipaq.ki.se), and vigorous physical activity was multiplied by two. The physical activity time was categorized into two groups: 0–150 min per week (defined as insufficient physical activity) and ≥ 150 min per week (defined as sufficient physical activity). This categorization followed physical activity guidelines [20] and was based on previous studies [18].

Other variables included age groups in complete years (60–69, 70 or more); sex (male or female); self-reported race/skin color (white, black or brown, yellow, indigenous or others); marital status (with a partner or without a partner); and education level, categorized by years of completed study (0–3, 4–7, 8–11, 12 or more).

The variables sex, age, race/skin color, and marital status were used to characterize and stratify the population studied in both surveys. The analyses were conducted using a single combined dataset containing records with information on all the studied variables, ensuring the comparability of results. Comparability was further verified through the calculation of prevalence and 95% Confidence Intervals (95%CI), which were estimated separately from the database of each survey. The variables were renamed and subsequently classified for database matching with identical values and labels for the same response category. A new variable was created to identify the source of the information, enabling comparisons.

For the indicators selected for this study, prevalence and their respective 95%CI were estimated for sociodemographic variables for each year of the ISACapital survey. Additionally, the percentage change in the categories of physical activity was calculated to determine changes over time. The formula for calculating the percentage change is (Final Value – Initial Value) ÷| Initial Value |× 100.

The Prevalence Ratio (PR) and 95%CI were estimated using robust variance through univariate Poisson regression models to assess the magnitude of differences between the estimates obtained in the 2003 and 2015 surveys among individuals who met physical activity recommendations (sufficient level) according to sociodemographic characteristics. Poisson regression was chosen because of the study design and the high prevalence of the binary outcome variable (> 10%), which provided prevalence estimates with more conservative confidence intervals [21]. Moreover, estimates using multiple Poisson regression models were explored, adjusting for age range, education, sex, and race/skin color, as indicated in the literature [22]. A 5% significance level was adopted for all tests.

Participants with incomplete information in the 2003 and 2015 surveys were treated as missing data.

Data analysis was performed using the survey via Stata (version 14, StataCorp, College Station, Texas, USA), incorporating a complex sample design—strata, clusters, and weights.

## Results

The sociodemographic profile of the studied population is evidenced in Table 1. According to sex and age distributions, similarities were observed, with a predominance of women and individuals aged 60–69 years. Regarding self-reported race/skin color and marital status most individuals identified as white and reported having a partner.

**Table 1:** Distribution of the older adult population according to sociodemographic characteristics. ISA-Capital 2003–2015

Variables and Categories	2003		2015	
**Age range (years)**	N	% (95%CI)	N	% (95%CI)
60–69	474	51.0 (47.1–55.0)	573	57.2 (53.0–61.5)
70 or more	398	49.0 (45.0–52.9)	446	42.8 (38.5–47.0)
**Sex**				
Female	451	60.3 (56.5–64.0)	632	59.7 (56.9–62.4)
Male	421	39.7 (36.0–43.5)	387	40.3 (37.6–43.1)
**Marital status**^a;c^				
With partner	558	63.5 (59.2–67,7)	515	52.8 (48.6–57.0)
Without partner	294	36.5 (32.3–40,8)	500	47.2 (43.0–51.4)
**Race/skin color**^b**;**d^				
White	589	75.5 (71.5–79.6)	611	64.9 (60.1–69.7)
Black or brown	234	20.7 (17.1–24.2)	318	27.3 (22.9–31.7)
Yellow, Indigenous or others	27	3.8 (2.0–5.6)	82	7.8 (5.7–9.9)

2003—^a^20 missing; ^b^22 missing. 2015 – ^c^4 missing; ^d^8 missing

The physical activity profile of the study population is presented in Table 2. The prevalence of insufficiently active older adults was higher in the 2003 survey (79.5%) and remained elevated in 2015 (69.9%). However, a positive trend was observed, with an increase in the percentage of older adults classified as sufficiently active over the 12-year period.

**Table 2:** Distribution and percentage change of the older adult population according to physical activity. ISA-Capital 2003–2015

**Variable and Categories**	2003		2015		% ∆
	N	% (95%CI)	N	% (95%CI)	
**Physical activity**					
Insufficiently active	695	79.5 (74.9–84.1)	719	69.9 (65.8–73.9)	−12.1
Sufficiently active	176	20.5 (15.9–25.1)	298	30.1 (26.1–34.2)	46.8

2003—1 missing; 2015—2 missing; % ∆—% change

Physical activity levels according to sociodemographic characteristics across the two surveys are evidenced in Table 3. In the adjusted model, a significant increase in the prevalence of individuals with sufficient physical activity levels was observed from 2003 to 2015 surveys. This increase was consistent across age range, sex, marital status, education (years), and race/skin color variables. Among individuals aged 60–69, the prevalence was 1.43 times greater (PR = 1.43; 95%CI 1.10–1.86) in 2015 than in the same age group in 2003.

**Table 3:** Distribution of sufficient physical activity among older adults by sociodemographic characteristics and regression analysis (ISACapital 2003–2015)

Variables and Categories	2003		2015		Crude PR (95%CI)	Adjusted PR (95%CI)^b^
	**Physical activity level**					
	**Sufficiently active**^**a**^		**Sufficiently active**			
**Age range (years)**	N	**% (IC95%)**	**N**	**% (IC95%)**		
60–69	109	22,7 (18,1-28,1)	195	34,1 (29,0-39,6)	**1,50 (1,15-1,97)**	1.43 (1.10–1.86)
70 or more	67	18,1 (12,8–25.0)	103	24,8 (20,6-29,6)	1,37 (0,93-2,00)	1.35 (0.94–1.95)
**Sex**						
Female	76	18,4 (13,4-24,6)	163	26,3 (21,8-31,3)	1,43 (1,00-2,04)	1.35 (0.97–1.88)
Male	100	23,7 (18,6-29,7)	135	35,9 (30,7-41,4)	1,52 (1,15-2,00)	1.45 (1.10–1.91)
**Marital status**						
With partner	117	20,8 (16,3-26,2)	162	32,3 (27,5-37,4)	1,55 (1,17-2,06)	1.42 (1.08–1.88)
Without partner	56	20,0 (14,9-26,4)	135	27,8 (23,0-33,1)	1,39 (0,99-1,95)	1.36 (0.98–1.89)
**Education (Years)**						
Never até 3	60	15,1 (11,4-19,7)	136	24,1 (20,4-28,2)	1,59 (1,16-2,19)	1.53 (1.11–2.09)
4 to 7	65	19,5 (14,6-25,5)	42	30,1 (21,6-40,3)	1,54 (1,02-2,35)	1.48 (0.97–2.27)
8 to 11	27	22,6 (13,1-36,1)	58	35,1 (26,0-45,4)	1,55 (0,87-2,78)	1.42 (0.80–2.51)
12 or more	19	32,6 (20,5-47,6)	61	40,9 (33,0-49,3)	1,25 (0,78-2,00)	1.13 (0.72–1.79)
**Race/skin color**						
White	120	21,7 (17,1-27,1)	171	29,3 (25,0-34,1)	1,35 (1,02-1,78)	1.30 (0.99–1.72)
Black or brown	49	19,6 (13,3-28,1)	102	31.5 (25.5–38.2)	1,61 (1,05-2,46)	1.70 (1.12–2.59)
Yellow, Indigenous or others	7	18,5 (8,2-36,5)	23	32,5 (21,6-45,6)	1,75 (0,75-4,08)	1.60 (0.63–4.01)

^a^Reference Category

^b^Adjusted for Age range (years), sex, race/skin color, and Education (Years)

When comparing the corresponding categories from 2015 and 2003 surveys, an increase was observed in the prevalence of older adults classified as sufficiently active. Among males, the prevalence was 1.45 times greater (PR = 1.45; 95% CI 1.10–1.91); among those with a partner, the prevalence was 1.42 times greater 1.42 times greater (PR = 1.42; 95% CI 1.08–1.88); and among older adults with up to three years of education, 1.53 times greater (PR = 1.53; 95% CI: 1.11–2.09). Additionally, individuals who self-identified as black and brown regarding race/skin color showed a 1.70 times greater prevalence of sufficient physical activity (PR = 1.70; 95% CI 1.12–2.59), Table 3.

## Discussion

This study estimated a 46.8% increase in the prevalence of older adults achieving sufficient physical activity levels over 12 years. A comparison of sociodemographic categories between the 2003 and 2015 surveys revealed an increase in sufficient physical activity among individuals aged 60–69, males, those with a partner, with up to three years of education, and those who self-identified as black and brown in terms of race and skin color. In contrast, the prevalence of sufficient physical activity remained stable among females without partners, those with more than four years of education, and those of race/skin colors: white, yellow, indigenous, or others.

Contrary to global data [23] and findings from developed countries [24], this study revealed an increase in older adults meeting sufficient physical activity standards, supporting the findings of a Brazilian study [15]. A survey with a representative sample of older adults living in capital cities reported an increase from 23.3% in 2009 to 27.5% in 2020 [15]. In contrast, the results from the U.S. health survey revealed an increase in physical activity in leisure-time domains and stability in transportation domains [25]. Despite the observed progress, both nationally and municipally, approximately 70% of the population remains below the recommended physical activity levels for health, underscoring the need for interventions to promote regular physical activity to achieve the World Health Assembly’s goal of reducing insufficient physical activity by 15% between 2010 and 2030 [26].

This increase in sufficient physical activity among older adults may be partially explained by the implementation and expansion of public policies and health promotion programs during the period analyzed. For example, initiatives like the Programa Saúde da Família and community-based physical activity programs like Academia da Cidade, later expanded under the Academia da Saúde initiative, likely contributed to increasing access and opportunities for physical activity in public spaces. Additionally, the dissemination of physical activity guidelines, growing public awareness of healthy aging, and the inclusion of physical activity promotion in primary care initiatives may have influenced the behavior of older adults [27]. These factors, in combination with broader social and cultural shifts valuing active aging, may help explain the observed increase in physical activity levels.

When changes over the 12-year period were analyzed by age group, the study revealed that older adults aged 60 to 69 showed approximately 43% increases in the prevalence of sufficient physical activity. These results are similar to a survey conducted at the national level in Brazil, such as those of Soares et al., who reported a significant increase in physical activity among older adults aged 69 years and younger [15]. The availability of spaces such as parks, squares, and bike lanes in the city of São Paulo likely contributed to this observed phenomenon. However, the literature suggests that insufficient physical activity tends to increase with age [23, 24, 28].

When comparing data from 2003 and 2015, males showed a 45% increase in the prevalence of sufficient physical activity. Global results and findings from some countries have indicated that the prevalence of sufficient physical activity is greater among men than women [15, 23–25]. This trend aligns with previous evidence suggesting that men with higher educational levels are more physically active during leisure-time [29]. Several factors may have contributed to this increase, specifically among older men. Cultural norms and gender roles may continue to favor men's participation in recreational or structured physical activities.

Additionally, recent literature has suggested that education level is a significant determinant of physical activity. In the present study, there was stability in the prevalence of sufficient physical activity across the categories of education variables, similar to a study conducted in Brazil [15]. A time series study conducted between 2009 and 2020 with older adults Brazilians identified an increase in the prevalence of leisure-time physical activity among both males and females, including those who reported having 0–8 years of education [15].

From 2003 to 2015, the prevalence of sufficiently active individuals among older adults with a partner increased by 1.42 times, a finding that contrasts with previous studies [15, 18, 30]. Ferreira et al*.* observed a higher prevalence of sufficient physical activity among older adults with multimorbidity who did not have a partner [18]. In contrast, Soares et al. reported increase physical activity levels among both married individuals and those not in a stable relationship [15]. These divergent findings suggest that the relationship between marital status and physical activity in older adults may be influenced by contextual or cohort-specific factors that warrant further investigation.

Notably, the influence of education level on physical activity can be explained by a greater understanding of its health and well-being benefits, better knowledge of exercise types, and increased access to such activities. On the other hand, considering the tendency for lower income among individuals with less education, economic constraints may result in a lack of resources for engaging in physical activities, which could increase physical activity in transportation.

Regarding race/skin color, the results of this study indicate that, over 12 years, the prevalence of sufficient active individuals who identified as black and brown increased 1.70 times. Notably, in the crude model, the prevalence of sufficient physical activity among those identified as white was 35% higher in the 2015 survey than in the same group in the 2003 survey. Similar to the present study, extensive evidence has revealed differences in physical activity prevalence across race/skin color categories [31]. However, no studies to date have explored differences within racial groups themselves.

Some limitations should be considered regarding this study. Laboratory and/or clinical tests are not used to assess or measure physical activity levels, as these procedures are costly and time-consuming. This limitation may have contributed to the overestimation of the results. A cognitive screening was not applied, and the International Physical Activity Questionnaire (IPAQ) was used, which may be subject to recall bias, especially among older adults. Additionally, we did not exclude individuals with chronic diseases or disabling conditions from the analyses. While such conditions may influence participation in physical activity, we aimed to assess patterns and inequalities in physical activity among the general older adult population, including those with health-related limitations. Nonetheless, we acknowledge that severe morbidity could have influenced our findings, which should be considered when interpreting the results. The exclusion of institutionalized and homeless older adults may have led to an underestimation of health and social inequalities. In this study, we do not include potential confounding variables such as income or access to public facilities for physical activity, as these were not consistently available across survey years. Future studies employing a causal or explanatory approach should consider incorporating these factors to better understand the mechanisms underlying observed inequalities.

However, the method used to measure physical activity has strengths, such as using the controlled version of the IPAQ, a validated and widely used instrument worldwide. This tool allows independent assessment of different physical activity domains (leisure-time and transportation), supports comparisons with other studies, ensures good participant acceptability, and enables respondents to recall their activity from the week before the interview, which allows interviewees to recall their physical activity practices. Although post-stratification weighting helped to correct for differential response patterns across age and sex groups, we did not apply specific statistical techniques to assess or adjust for non-response bias. This should be considered when interpreting the findings. ISA-Capital is a health survey with a representative sample of the older adult population in the city of São Paulo; therefore, the results can only be generalized to older adults residing in the city of São Paulo, Brazil.

## Conclusions

In conclusion, the results indicate an increase in the prevalence of older adults meeting sufficient physical activity levels over 12 years, although this increase is unequally distributed. This inequality is reflected in the differences in the prevalence of sufficient physical activity across sociodemographic characteristics, emphasizing the need for a deeper understanding of the barriers that may hinder the implementation of successful interventions to promote physical activity among the most vulnerable groups.

## Data Availability

The datasets used and/or analyzed during the current study are available from the corresponding author on reasonable request.
